# Cysteine redoxome landscape in mouse brown adipose tissue under acute cold exposure

**DOI:** 10.1016/j.isci.2025.112051

**Published:** 2025-02-17

**Authors:** Hein Ko Oo, Cynthia M. Galicia-Medina, Takumi Nishiuchi, Ryota Tanida, Hisanori Goto, Yujiro Nakano, Yumie Takeshita, Yoshiro Saito, Hiroaki Takayama, Toshinari Takamura

**Affiliations:** 1 Department of Endocrinology and Metabolism, Kanazawa University Graduate School of Medical Sciences, Kanazawa, Japan; 2 Division of Natural System, Kanazawa University Graduate School of Natural Science and Technology, Kanazawa, Japan; 3 Laboratory of Molecular Biology and Metabolism, Graduate School of Pharmaceutical Sciences, Tohoku University, Sendai, Japan; 4 Life Sciences Division, Engineering and Technology Department, Kanazawa University Graduate School of Medical Sciences, Kanazawa, Japan

**Keywords:** Physiology, Cell biology, Functional aspects of cell biology, Proteomics

## Abstract

Reversible cysteine post-translational modifications serve as a "switch" for protein structure-function dynamics. While reversible cysteine oxidation in uncoupling protein 1 is known to play a role in brown fat thermogenesis, the full cysteine redoxome affected by cold exposure remains unexplored. We established a strategy for comprehensively mapping the cysteine redoxome by pinpointing oxidized and reduced cysteine residues in the brown adipose tissue of mice under room temperature and acute cold exposure. We identified over 1,000 labeled cysteine residues under room and cold temperatures. Cold exposure shifted the cysteine redox states toward oxidation. Cold-sensitive reactive cysteine residues were enriched in biological processes and molecular functions associated with thermogenesis pathways. The presence of proximal positively charged and negatively charged amino acids determined the highly reactive and non-reactive cysteine residues, respectively, under cold exposure. Our findings broaden the landscape of cold-sensitive proteome and identify potential therapeutic targets to fine-tune thermogenesis.

## Highlights

• Alkylation-based redoxome pinpoints both oxidized and reduced cysteine residues
• Cold exposure perturbs cysteine redox status in brown fat
• Cold-reactive cysteines are enriched in mitochondrial activation pathways
• Nearby positively charged amino acids enhance cysteine redox reactivity

## Graphical abstract

**Figure:**
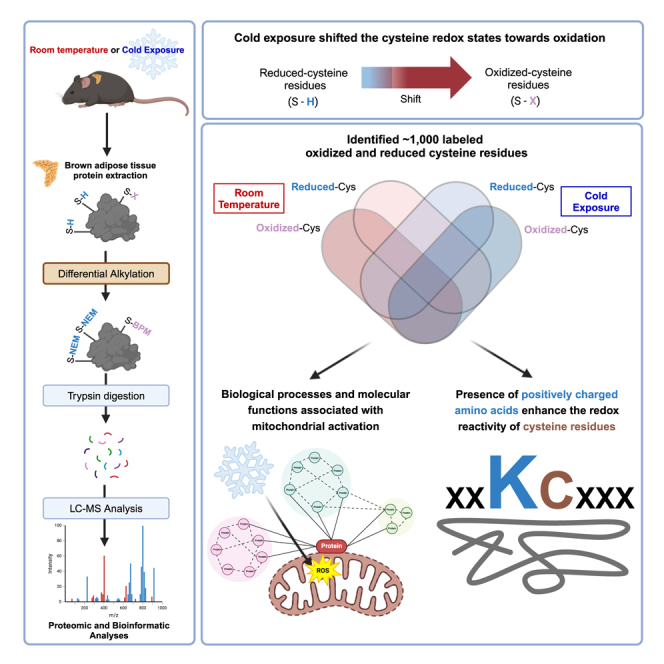

## Introduction

Activating brown adipose tissue (BAT) to convert excess energy into heat has emerged as a promising approach to combating obesity and diabetes.^1^^,^^2^ BAT, a specialized adipose tissue, primarily dissipates chemical energy as heat through non-shivering thermogenesis.^1^ The thermogenic activity of BAT is typically initiated by cold-induced sympathetic activation, triggering the release of noradrenaline (NA) and subsequent β3 adrenergic signaling, which activates uncoupling protein 1 (UCP1). Accumulating evidence from animal and human studies indicates that BAT’s thermogenic capacity plays a crucial role in whole-body energy expenditure, metabolic homeostasis, and substrate utilization.^3^^,^^4^^,^^5^ When fully activated, BAT serves as a significant metabolic sink, aiding in the clearance of glucose, triglycerides, and other specific metabolites from circulation, thereby decreasing serum glucose and lipid levels.^6^^,^^7^

The amino acid cysteine (Cys) can adopt various oxidation states, imparting structural flexibility to proteins due to its sulfur atom, which can be switched between reduced (thiol) and oxidized forms in response to redox fluctuations.^8^ Oxidized cysteine residues encompass reversible cysteine oxidative post-translational modifications (Cys-PTMs) such as disulfide bonds (S-S), S-sulfenylation (S-OH), S-nitrosylation (S-NO), and S-glutathionylation (S-SG). Reactive oxygen species (ROS) play a dual role, serving as crucial signaling molecules at optimal concentrations while potentially causing damage at higher levels.^9^ Indeed, ROS can influence redox signaling, protein structure, and function through reversible oxidation of Cys-PTMs.^10^^,^^11^^,^^12^ In this context, ROS can modulate protein structures and functions by altering the redox state of cysteine residues, acting as binary “switches.”

Cysteine redox switches play a crucial role in diverse metabolic processes. Research has shown that S-nitrosylation of insulin receptor β subunit, insulin receptor substrate 1, and protein kinase B/Akt is implicated in the development of insulin resistance.^13^ Additionally, reversible S-glutathionylation of proteins involve in the regulation of glucose metabolism^14^ and contributes to various cardiovascular disorders.^15^ Furthermore, both reversible disulfide bond formation and S-nitrosylation have been found to modulate ATP synthase function.^16^

Cold exposure activates the sympathetic nervous system, enhances the NA release from the nerve endings, and upregulates lipolysis and fatty acid oxidation via adrenergic signaling in brown adipocytes.^17^^,^^18^ The increased fatty acid catabolism enhances mitochondrial respiration and electron leakage from the electron transport chain and produces ROS.^17^ The mitochondria-derived ROS change the redox state of the cysteine residue of UCP1.^19^^,^^20^ The mitochondrial ROS-mediated oxidation of Cys^254^ of UCP1 (UCP1 sulfenylation) is crucial for thermogenesis. Indeed, antioxidants eliminate ROS, significantly inhibit thiol oxidation, and impair cold tolerance.^18^^,^^19^^,^^21^ Previously, we reported that diabetes-related elevation of selenoprotein P reduces Cys^254^ of UCP1 through glutathione peroxidase 4, impairing thermogenesis in BAT.^18^ These findings led us to hypothesize that cysteine residues in other proteins may also be reversibly oxidized or reduced under cold exposure, contributing to dynamic metabolism in BAT and promoting thermogenesis.

In this study, we developed a straightforward method to assess the cysteine redox landscape by detecting both oxidized and reduced Cys-PTMs in the BAT of mice exposed to acute cold. Our goal was to identify specific cold-sensitive cysteine residues and their associated pathways, as well as potential motifs influenced by cold exposure. These findings could lead to novel therapeutic targets for diabetes and obesity.

## Results

### Mapping the cysteine redoxome and proteome in the brown adipose tissue of mice under room temperature or cold exposure

**Figure 1:**
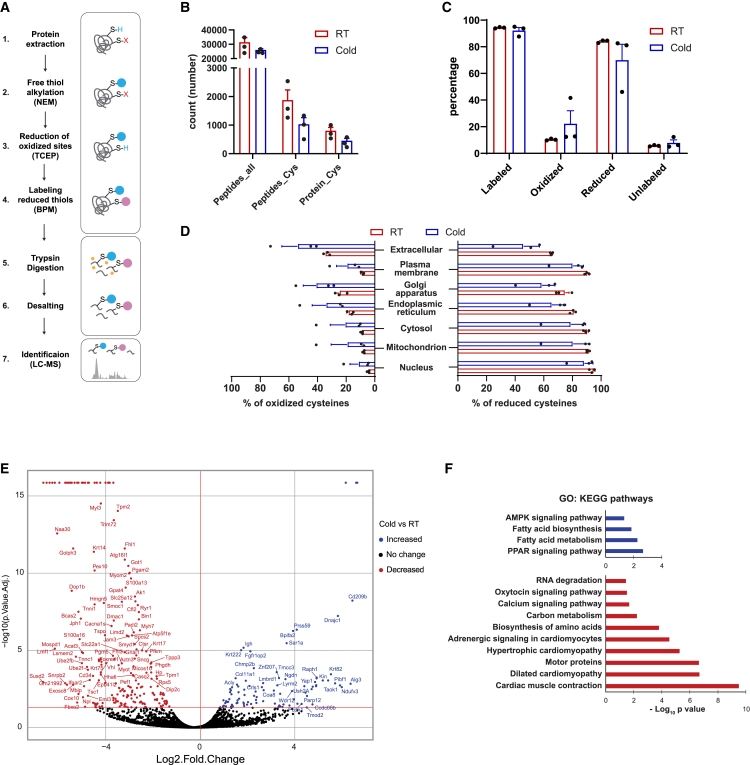
Cysteine redoxome profile in mouse brown adipose tissue under RT and cold conditions (A) Differential alkylation-based bioswitch labeling workflow: (1) Protein extraction from brown adipose tissues, (2) alkylation of native thiol groups (represented as S–H) on cysteines using n-ethylmaleimide (NEM, represented in a blue circle), (3) reduction of reversibly oxidized post-translational modifications (represented as S-X) on cysteines with tris(2-carboxyethyl) phosphine (TCEP), (4) labeling of nascent thiol groups (represented as S-H) with biotin-peac5-maleimide (BPM; represented in a red circle), (5) trypsin digestion of proteins to generate labeled and unlabeled peptides, (6) high pH reverse-phase peptide fractionation to desalt proteins and remove impurities, and (7) LC-MS analysis of peptides to identify total cysteine residues in the sample. (B) Number of all peptides, cysteine containing peptide and cysteine containing protein in three replicates. (C) Percentage (%) of all quantified reduced or oxidized cysteine residues in three replicates. (D) Subcellular distribution of oxidized and reduced cysteine residues in BAT in three replicates. (E) Volcano plot depicting changes in protein abundance between cold vs. RT conditions. Data derived from triplicated proteomic analyses of BAT under cold and RT conditions. The horizontal red line indicates a *p*-value of 0.05, while the vertical red line marks a fold change of 0, indicating significantly increased or decreased proteins. (F) Top enriched KEGG pathways of proteins significantly increased and decreased based on the volcano plot (E). The protein abundances and complete list of enriched KEGG metabolic pathways can be found in Tables S1, S2, and S3. In B–D, data are represented as mean ± SEM. Statistical significance is calculated by two-tailed unpaired Student’s t test. There is no significant difference between RT and Cold groups in B–D.

We investigated the cysteine redoxome profile in the BAT of mice exposed to acute cold (3 h at 4°C) compared to a room temperature (RT) control group, composed of 3 mice in each group. We utilized a differential alkylation-based proteomics technique without denaturing agents (Figure 1A). In the RT group, we detected about 30,000 peptides with 1,800 Cys-containing peptides belonging to about 800 proteins. In the cold group, we detected about 26,000 peptides with 1000 Cys-containing peptides belonging to about 500 proteins (Figure 1B). Cysteine residues were categorized as follows: those labeled with N-ethyl-maleimide (NEM) indicating reduced cysteine; those labeled with biotin-PECA5-maleimide (BPM) indicating oxidized state (reversibly oxidized cysteine, reduced by a reducing agent), and unlabeled cysteine residues. Only 5.6% and 7.8% of cysteine residues in the RT and cold groups, respectively, were unlabeled (Figure 1B), demonstrating the efficiency of the differential alkylation method in labeling these sites; in both cases, over 90% of cysteine residues were labeled according to their redox status. The cold group tended to exhibit a higher percentage of oxidized cysteines compared to the RT group (22.2% vs. 10.3% respectively) (Figure 1B).

To investigate the subcellular distribution of proteins with quantified cysteine residues, we categorized proteins based on their oxidized and reduced cysteine states using a subcellular localization database under room temperature (RT) and cold conditions (Figure 1C). Across all compartments, nucleus, mitochondrion, cytosol, endoplasmic reticulum, Golgi apparatus, plasma membrane, and extracellular space, the percentage of proteins with reduced cysteine exceeded those with oxidized cysteines under both temperature conditions.

Under cold exposure, a tentative shift toward a more oxidizing environment was observed across cellular compartments, characterized by changes in the redox state of cysteine residues (Figure 1D). Compared to RT, the cold group exhibited an increase in the percentage of oxidized cysteine residues in extracellular space (19.2%), followed by the Golgi apparatus (16.15%), endoplasmic reticulum (15.56%), cytosol (11.5%), mitochondria (11.19%), plasma membrane (10.3%), and nucleus (6.67%).

Next, we identified the protein abundances under RT and cold exposure, plotted them in a volcano plot (Figure 1E; Table S1), and mapped the significantly increased or decreased proteins to Kyoto Encyclopedia of Genes and Genomes (KEGG) pathways (Figure 1F; Tables S2 and S3). The proteins upregulated in the cold exposure group were enriched in pathways such as the PPAR signaling pathway, fatty acid metabolism, and the AMPK signaling pathway. Conversely, the downregulated proteins were associated with various biological processes, including cardiac muscle contraction and myopathy, adrenergic signaling, carbon metabolism, amino acid biosynthesis, calcium signaling, oxytocin signaling, and RNA degradation.

### Temperature-sensitive cysteine redoxome identification and network of metabolic pathways in the brown adipose tissue

**Figure 2:**
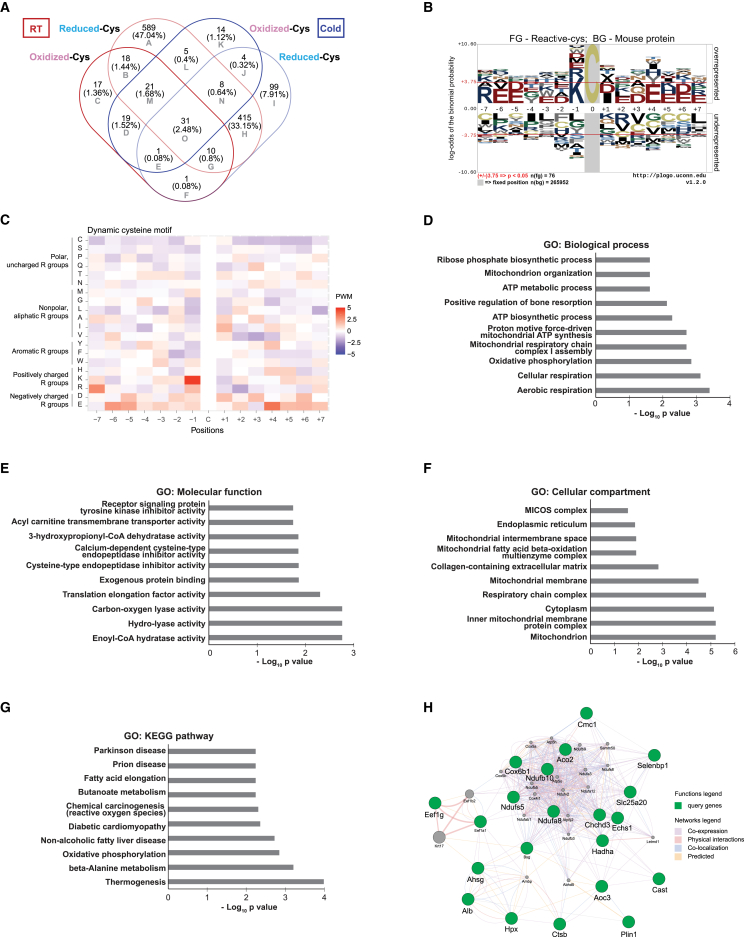
Identifying dynamic cysteines along with GO terms, KEGG enrichment analysis, and functional prediction (A) Venn diagram depicting overlap and exclusivity of reproducible oxidized and reduced cysteine residues validated in triplicate experiments under RT and Cold as shown in Figure S1. Cysteine residues in Regions L, M, and N are denoted as the cold-reactive cysteine residues. Region L – reproducible cysteine residues found reduced under RT and oxidized under cold; Region M – reproducible cysteine residues found reduced & oxidized under RT and oxidized under cold; Region N – reproducible cysteine residues found reduced under RT and reduced & oxidized under cold. (B) Sequence motif analyses of dynamic cysteine residues, examining proximal positions (± seven positions) relative to the cysteine sites. Horizontal red lines indicate significance at a *p*-value of 0.05. (C) Heatmaps illustrating amino acid sequences surrounding dynamic cysteine sites (B). The heatmaps display position weight matrix (PWM) values, reflecting amino acid frequencies proximal to the cysteine and categorized by amino acid properties. (D–F) GO enrichment analyses based on BPs (D), MFs (E), and CCs (F) observed in reactive cysteine residues in BAT under RT and Cold groups. Selected terms relevant to cellular homeostasis are highlighted. Comprehensive lists of enriched terms are available in Tables S4, S5, and S6. (G) Ten relevant enriched KEGG pathways identified in reactive cysteine residues in BAT under RT and Cold groups. The complete list of enriched KEGG metabolic pathways can be found in Table S7. (H) Function prediction of proteins containing reactive cysteine residues using GeneMANIA. The legend within the network illustrates the type of connections between genes/proteins.

Next, we identified reproducible cysteine sites by selecting those detected in at least two independent replicates per experimental condition (Figure S1). Our results were validated by comparison with previously published sites of Cys-PTMs (Figure S2). We also examined the biotinylation status of MIC19, as a representative, showing an apparent increase in cysteine oxidation, though it didn’t reach statistical significance (Figure S3). The reproducible cysteine residues included 189 oxidized and 1097 reduced sites in the RT group, and 103 oxidized and 569 reduced sites in the cold group, illustrated in a Venn diagram (Figure 2A). Most proteins containing reproducible cysteine residues showed no significant change in abundance between RT and cold (data not shown). We specifically identified 76 dynamic cysteine residues that were reversibly oxidized or reduced after cold exposure, accounting for approximately 6% of reproducible cysteines (Region E, G, L, M, N, and O, Figure 2A). To explore the amino acid motifs surrounding these dynamic cysteine residues, we aligned peptide sequences within a ±7 amino acids window proximal to the cysteine and conducted motif analysis. The findings are depicted in pLogo plots and heat maps, presenting the position weight matrix for each amino acid based on its properties (Figures 2B and 2C). Previous studies have highlighted the role of proximal charged amino acids in encoding cysteine redox sensitivity,^10^^,^^11^^,^^12^ indicating that cysteine residues near positively charged amino acids, such as lysine (Lys, K), are more likely to exist in a thiolate state (-S^-^, which is an excellent nucleophile) and are more susceptible to oxidation. Indeed, our analysis reveals significant enrichment of positively charged amino acid lysine at the −1 position of dynamic cysteine sites (Figures 2B and 2C).

We focused on 34 dynamic cysteine residues particularly susceptible to oxidation under cold exposure, categorizing them as reactive cysteine residues (Regions L, M, and N in Figure 2A; Table 1). Using the MUSCLE algorithm, we conducted multiple sequence alignments to assess the conservation of these cysteine residues across human and bovine protein sequences. Our analysis revealed that these critical cysteine residues are predominantly conserved between human and bovine sequences (Figure S4). We further explored the significance of these reactive cysteine residues in metabolism through gene ontology (GO) and KEGG enrichment analyses. GO enrichment analyses identified 59 biological processes (BPs) (Table S4), including cellular respiration, mitochondrial respiratory chain complex I assembly, ATP synthesis, ATP metabolism, and mitochondrion organization (Figure 2D). Additionally, 39 molecular functions (MFs) (Table S5) were identified, such as enoyl-CoA hydratase activity, hydro-lyase activity, carbon-oxygen lyase activity, translation elongation factor activity, exogenous protein binding, cysteine-type endopeptidase inhibitor activity, calcium-dependent cysteine-type endopeptidase inhibitor activity, 3-hydroxy propionyl-CoA dehydratase activity, acylcarnitine transmembrane transporter activity, and receptor signaling protein tyrosine kinase inhibitor activity (Figure 2E). Moreover, 47 cellular compartments (CCs) (Table S6) were enriched, encompassing mitochondria matrix, inner membrane, respiratory chain, and mitochondrial contact site, and cristae organizing system (MICOS) complex (Figure 2F).

**Table 1:** List of reactive cysteine residues

Condition	Accession ID	Gene Symbol	Protein Name	Cysteine Position	Subcellular localization
Reduced in RT and Oxidized in Cold exposure (Region L in Figure 2A)	Q9DCS9	Ndufb10	NADH dehydrogenase 1 beta subcomplex subunit 10	77	Mitochondria, Cytosol
	Q9CRB9	Chchd3	MICOS complex subunit Mic19	183	Nucleus, Mitochondria, Cytosol
	P10126	Eef1a1	Elongation factor 1-alpha 1	411	Nucleus, Cytosol, Plasm membrane
	A0A1S6GWI0	Ndufa8	NADH dehydrogenase 1 alpha subcomplex subunit 8	70	Mitochondria, Cytosol
	P17563	Selenbp1	Selenium-binding protein 1	8	Nucleus, Cytosol
Oxidized or reduced in RT; Oxidized in Cold exposure (Region M in Figure 2A)	Q91X72	Hpx	Hemopexin	230	Extracellular space
	P18572	Bsg	Basigin	203	Cytosol, Endoplasmic reticulum, Plasma membrane
	P29699	Ahsg	Alpha-2-HS-glycoprotein (Fetuin-A)	219	Cytosol, Golgi apparatus, Extracellular space
	Q9CVE7	Cmc1	COX assembly mitochondrial protein	57	Mitochondria, Cytosol
	O70423	Aoc3	Membrane primary amine oxidase	430	Cytosol, Endoplasmic reticulum, Golgi apparatus, Plasma membrane, Extracellular space
	Q8CGN5	Plin1	Perilipin-1	55	Cytosol, Endoplasmic reticulum
	Q921I1	Tf	Serotransferrin	28, 67, 260, 363	Extracellular space
	A0A1S6GWI4	Ndufs5	NADH dehydrogenase iron-sulfur protein 5	108	Mitochondria, Cytosol
	Q546G4	Alb	Albumin	77, 86, 461, 462, 500, 501, 511	Cytosol, Endoplasmic reticulum, Golgi apparatus, Extracellular space
	P10605	Ctsb	Cathepsin B	93	Mitochondria, Cytosol, Plasma membrane, Extracellular space
	A0A1S6GWI0	Ndufa8	NADH dehydrogenase 1 alpha subcomplex subunit 8	144	Mitochondria, Cytosol
	P56391	Cox6b1	Cytochrome *c* oxidase subunit 6B1	54	Mitochondria, Cytosol
Reduced in RT; Oxidized or reduced in Cold exposure (Region N in Figure 2A)	Q8BMS1	Hadha	Trifunctional enzyme subunit alpha, mitochondrial	470	Mitochondria, Cytosol
	Q7TPW6	Slc25a20	Solute carrier family 25 (Mitochondrial carnitine/acylcarnitine translocase)	155	Mitochondria, Cytosol
	Q921I1	Tf	Serotransferrin	386, 395	Extracellular space
	Q99KI0	Aco2	Aconitate hydratase, mitochondrial	126	Mitochondria, Cytosol
	P51125	Cast	Calpastatin (Calpain inhibitor)	408	Cytosol, Endoplasmic reticulum
	Q9D8N0	Eef1g	Elongation factor 1-gamma	266	Nucleus, Cytosol
	Q8BH95	Echs1	Enoyl-CoA hydratase, mitochondrial	111	Mitochondria, Cytosol

The KEGG enrichment analysis of reactive cysteine residues unveiled 25 key pathways, highlighting critical metabolic processes and disease associations (Table S7). Among these, the most significantly enriched pathways included thermogenesis, beta-alanine metabolism, and oxidative phosphorylation, which are closely linked to energy metabolism and mitochondrial function (Figure 2G). Additionally, pathways associated with diseases such as non-alcoholic fatty liver disease (NAFLD), diabetic cardiomyopathy, chemical carcinogenesis, prion disease, and Parkinson’s disease were enriched. Moreover, pathways related to lipid metabolism, such as butanoate metabolism and fatty acid elongation, were also enriched, suggesting alterations in lipid processing. These findings collectively offer insights into the fundamental biological processes and potential disease mechanisms influenced by our identified reactive cysteine residues.

Next, the protein association network, constructed using Cytoscape 3.10.2 and the GeneMania plugin 3.5.3, revealed significant interconnectivity among proteins containing reactive cysteine residues, particularly those associated with mitochondria. These proteins showed strong associations in terms of co-expression, physical interaction, and co-localization (Figure 2H), implying that their reactive cysteine residues likely coordinate cellular processes and respond to cold stress.

Further analysis of the reactive cysteine residues was conducted to elucidate their intramolecular localization and properties (Table S8) compared to the non-reactive cysteines exclusively reduced under cold exposure (Table S9). Of the 34 identified reactive cysteine residues, 15 are in loop regions, 15 in alpha-helical structures, and 5 in beta-sheet conformations within the protein (Figure S5A). Notably, 26 cysteine residues were found to participate in intramolecular disulfide bond formation (Figure S5B), exhibiting dihedral angle energies (DAE) ranging from approximately 3 to 22 kJ/mol (Figure S5C). Research has shown that disulfide bonds with higher degrees of DAE are more susceptible to destabilize.^22^^,^^23^ The mean pKa value for non-disulfide reactive cysteine residues was approximately 10, with no difference between reactive and non-reactive cysteine residues (Figure S5D). In addition, the PROPKA 3 analysis revealed that non-reactive cysteine residues were buried inside the protein structure compared to the reactive cysteine residues (Figure S5E). These findings provide insights into the structural distribution and chemical characteristics of reactive cysteine residues within the protein, with implications for their functional roles and potential redox sensitivity.

### Distinct motifs for exclusively reduced cysteine residues in room temperature and cold exposure

**Figure 3:**
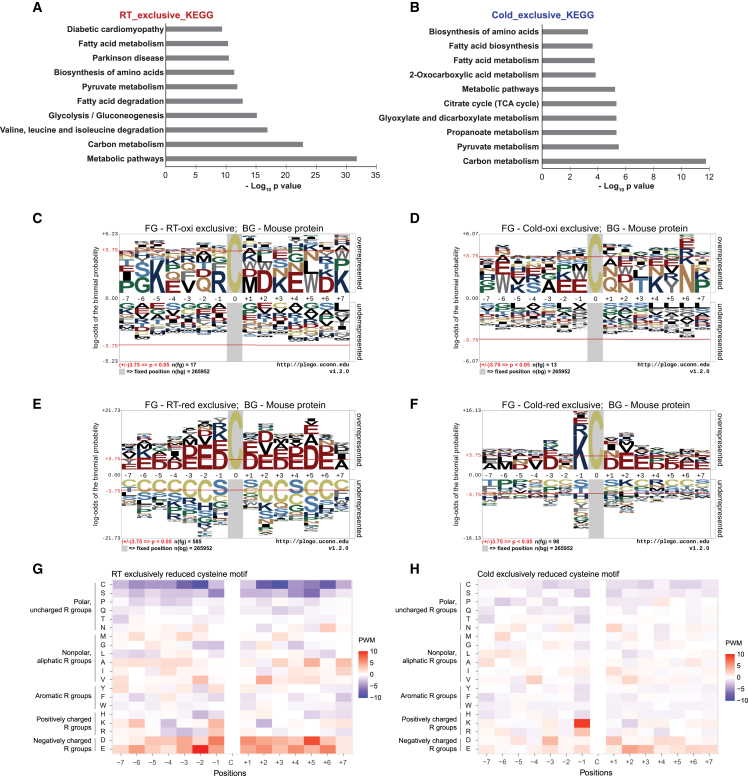
Exclusive cysteine redoxome analysis in RT and Cold groups with KEGG and motif analysis (A and B) We identified ten relevant enriched KEGG pathways associated with exclusive cysteine residues in BAT under RT (A) and cold (B). The complete list of enriched KEGG metabolic pathways is presented in Tables S16 and S17. (C–F) Sequence motif analyses were conducted for exclusively oxidized cysteine residues in RT (C), exclusive oxidized cysteine residues in Cold (D), exclusive reduced cysteine residues in RT (E), and exclusive reduced cysteine residues in Cold (F), spanning proximal positions (±seven). Horizontal red lines denote a significance level of *p*-value 0.05. (G and H) Heatmaps illustrating amino acid sequences surrounding reduced cysteine sites in RT (G) and Cold (H). These heatmaps depict the position weight matrix (PWM) values reflecting amino acid frequencies proximal to cysteine, categorized by amino acid properties.

Subsequently, we explored the significance of exclusive cysteine residue sites in each condition, combining those uniquely involved in the RT and cold groups (Regions A-C and I-K, respectively, in Figure 2A), and performed GO and KEGG enrichment analyses. GO enrichment analyses revealed similar enriched terms in both conditions: carboxylic acid, acetyl-CoA, and purine nucleotide metabolic processes in BPs; catalytic activity, nucleotide binding, and anion binding in MFs; and cytosol and mitochondria in CCs for both RT and cold exclusive cysteine residues (Figure S6; Tables S10, S11, S12, S13, S14, and S15). The KEGG analysis identified 63 and 20 enriched pathways in the RT and cold groups, respectively. Among these, the top relevant enriched pathways included metabolic pathways, carbon metabolism, pyruvate metabolism, fatty acid metabolism, and amino acid synthesis, showing similarities between the RT and cold groups (Figures 3A and 3B; Tables S16 and S17).

We analyzed peptide motifs associated with exclusive oxidized or reduced cysteine residues to gain deeper insights. While the oxidized cysteine residues did not exhibit significant motifs (Figures 3C and 3D), the reduced cysteine residues displayed distinct patterns (Figures 3E and 3F) contrasting with the dynamic cysteine residues. Specifically, in the RT group, exclusively reduced cysteine residues were surrounded by negatively charged amino acids such as aspartic acid (Asp, D) and glutamic acid (Glu, E) at positions −3, −2, −1, 1, and 2 (Figures 3E and 3G). In the cold group, although the proximal presence of positively charged amino acid lysine (Lys) was observed at the −1 position, these reduced cysteine residues were surrounded by negatively charged amino acids (Asp and Glu) at positions −3, 2, and 3 (Figures 3F and 3H). These results suggest that the proximity of negatively charged amino acids may influence the reactivity of cysteine residues in proteins, potentially affecting their redox status.

## Discussion

The study has established an accessible method for labeling reversible cysteine residues and has detailed a cysteine redox profile encompassing over 1,000 oxidized and reduced cysteine residues in mouse BAT under RT and cold exposure conditions. Under acute cold exposure, there is a shift toward oxidation in the cysteine redoxome of BAT, increasing from 10.3% to 22.2% of total cysteine residues, which persists across all subcellular compartments. The cold-sensitive reactive cysteine residues demonstrate significant enrichment in mitochondrial respiration and thermogenesis pathways, as indicated by GO and KEGG analyses. Additionally, we identified distinct cysteine motifs undergoing dynamic redox changes and others that remain exclusively reduced. Notably, the presence of proximal positively charged amino acids enhances the susceptibility of cysteine residues to cold-induced oxidation, while nearby negatively charged amino acids mitigate this cold sensitivity.

The study marks the first direct identification of reversibly reduced cysteine residues alongside reversibly oxidized ones, allowing for precise calculation of their percentages across all subcellular compartments under different temperature conditions. The endoplasmic reticulum is known to maintain a highly oxidative environment for effective protein folding.^24^ However, our findings reveal that a majority of reversible cysteine residues in various subcellular organelles, including the endoplasmic reticulum, are predominantly in the reduced state. This observation aligns with previous research that reported on the percentage of oxidized cysteine residues relative to total cysteine residues.^10^

Our findings indicate an overall oxidative shift in cysteine redox states during cold exposure, consistent with previous studies highlighting increased oxidative stress and redox imbalance under similar conditions.^18^^,^^19^ Interestingly, the oxidative shift varied notably among different subcellular compartments. Mitochondria are typically considered the primary target of oxidative stress induced by cold.^25^ Unexpectedly, our study revealed that cold exposure resulted in a limited increase in oxidized cysteine residues within mitochondria compared to other compartments. This finding, coupled with the high levels of reduced cysteine residues at RT, suggests that mitochondria maintain a predominantly reduced environment to protect themselves from oxidative damage. We observed a more pronounced oxidative shift in the endoplasmic reticulum compared to mitochondria. This may contribute to inter-organellar communications through lipid and protein trafficking involved in mitochondrial fission^26^^,^^27^ and cristae formation,^28^ enhancing mitochondrial activation in response to cold exposure. The highest oxidative shift was observed in extracellular proteins. Previous studies have reported that the Cys-PTMs were observed in key extracellular proteins of inflammation and viral infection.^29^ Indeed, one study reported that 2 h of cold exposure upregulate the inflammatory cytokines in brown adipose tissues.^30^ The highest oxidative shift of extracellular proteins might be associated with inflammation in brown adipose tissues under cold exposure. Also, Cys-PTMs in the extracellular matrix (ECM) proteins may play a vital role in BAT activation because ECM protein-mediated cell-to-cell contact may be crucial for cellular functions. Further studies may be needed to prove these hypotheses.

Positively charged amino acid lysine near dynamic cysteine residues, including cold-sensitive reactive cysteine residues, promotes their oxidation.^10^ This proximity facilitates the formation of a highly reactive thiolate state within the cysteine residues, increasing their susceptibility to oxidation, a finding consistent with prior research.^10^ Our study identified dynamic or reactive cysteine residues conserved in both human and bovine, characterized by a distinct motif surrounded by positively charged amino acids. These cysteine residues likely confer adaptive advantages in responding to acute environmental changes such as cold exposure. GO BPs and MFs enrichment analyses revealed that reactive cysteine residues were significantly enriched in processes related to metabolic homeostasis, energy production, and mitochondrial organization. Dysregulation of these BPs and MFs is implicated in the development and progression of metabolic disorders, cardiovascular diseases, and cancer. Additionally, KEGG enrichment analyses highlighted that these reactive cysteine residues are enriched in pathways associated with metabolic diseases such as diabetic NAFLD, cardiomyopathy, and Parkinson’s disease. These findings underscore the potential association of reactive cysteine residues with various pathological conditions.

To date, only Cys^254^ and Cys^305^ of UCP1 have been identified as targets of cold exposure in BAT.^19^^,^^31^ Our study further identifies 34 additional reactive cysteine residues that undergo reversible modification due to cold exposure in BAT. These cysteine residues likely contribute to cold-induced thermogenesis. For instance, Cys^183^ in MICOS complex subunit Mic19 was found to undergo reversible oxidation under cold conditions. Cys^183^ within Mic19 forms an intramolecular disulfide bond formation crucial for folding its coiled-helix-coiled-helix domain, essential for high-affinity binding to other MICOS subunits and stabilizing the MICOS complex.^32^ A stable MICOS complex is vital for mitochondrial cristae formation and BAT thermogenesis.^33^ Despite the reported importance of this cysteine residue, its role in cold-induced BAT metabolism through redox regulation remains to be fully elucidated. Moreover, we identified several mitochondrial proteins with reversibly oxidized cysteine residues under cold exposure. These proteins may also play roles in activating mitochondria, thereby promoting thermogenesis. Therefore, our findings suggest that these candidate reactive cysteines could offer new therapeutic avenues for enhancing BAT thermogenesis with future molecular studies.

Many studies have used biotin-maleimide to alkylate the oxidized cysteine residues and enriched them for the LC-MS analysis.^10^^,^^11^^,^^34^ Such an approach in combination with the tandem mass tag can provide the percent oxidation of specific cysteine residues. However, our study directly analyzed the biotin-labeled and NEM-labeled cysteine residues without streptavidin enrichment before LC-MS analysis. This method can detect oxidized and reduced cysteines directly, not as the percent oxidation. Although the number of cysteine residues that can be detected may greatly decrease without enrichment, the direct detection of both oxidized and reduced cysteines allows us to detect not only the oxidative stress but also the reductive stress cysteine targets under cold exposure.

Previous studies,^10^^,^^11^^,^^34^ including the current one, face a common challenge in accurately quantifying reversibly oxidized cysteine residues, particularly S-persulfidation (S-SH). The alkylating agent N-ethylmaleimide (NEM) can react with the thiol group in S-persulfidated cysteines (S-S-NEM), leading to their misidentification as reduced cysteines (S-NEM) instead of oxidized.^35^ Future cysteine redoxome profiling in BAT should employ selective labeling techniques to mitigate this issue and ensure accurate identification.

In summary, our redoxome profiling technique broadens the scope of the cysteine proteome, allowing for the identification of potential therapeutic targets for metabolic diseases. Current therapies targeting oxidative stress have encountered challenges in conditions such as cancer,^36^ fatty liver disease,^37^ and type 2 diabetes.^38^ Understanding the dynamics of cysteine reduction and oxidation, including their specific motifs influenced by environmental temperature, could facilitate the development of targeted therapies to optimize BAT thermogenesis. This may contribute to the advancement of therapeutic drugs for combating obesity and diabetes.

### Limitations of the study

In this study, we mapped the cysteine redoxome of BAT under acute cold exposure and identified the cysteine residues that are cold-reactive and involved in mitochondrial thermogenic pathways. Further structural and functional analyses are needed to elucidate the impact of these cysteine modifications on protein functions.

## Resource availability

### Lead contact

Further information and requests for resources and reagents should be directed to and will be fulfilled by the lead contact, Toshinari Takamura (ttakamura@med.kanazawa-u.ac.jp).

### Materials availability

This study did not generate new unique reagents.

### Data and code availability

• The liquid chromatography-mass spectrometry proteomics data have been deposited to the jPOST^39^ (https://repository.jpostdb.org/), and the accession number was provided in the key resources table.
• This paper does not report the original code.
• Any additional information required to reanalyze the data reported in this paper is available from the lead contact upon request.

## Acknowledgments

We thank M. Kawamura and M. Togashi for technical support, Eng. Carlos A. Castro and Eng. Omar Bringas for technical support in utilizing bioinformatic tools and language programs, and Prof. Nathalie Grandvaux and Dr. Natalia Zamorano for methodological support. The graphical abstract is created in https://BioRender.com. This work was supported by the following grants: Japan Society for the Promotion of Science (JSPS) Grants-in-Aid for Scientific Research (KAKENHI) grant 23KF0035 (T.T and H.K.O). 10.13039/100007449Takeda Science Foundation (T.T).

## Author contributions

H.K.O. conceptualized the project, designed experiments, coordinated research, conducted bioinformatic analyses, interpreted data, and wrote the manuscript with input from all authors. C.M.G.M., and R.T. improved technical methodology, interpreted data, and discussed the data. T.N. performed mass spectrometry experiments and analyzed data. H.G., Y.N., and Y.T. interpreted data and supported the discussion. Y.S. supported the development of differential alkylation-based labeling and supported the data discussion. H.T. planned and designed experiments, coordinated research, and interpreted data. T.T. conceived the project, supervised the research, interpreted the data, provided discussion, and edited the manuscript.

## Declaration of interests

The authors declare no competing interests.

## STAR★Methods

### Key resources table

**Table:** 

REAGENT or RESOURCE	SOURCE	IDENTIFIER
**Antibodies**		

Rabbit anti-CHCHD3 polyclonal	Bethyl Laboratories	Cat# A305-497A, RRID: AB_2891424

**Chemicals, peptides, and recombinant proteins**		

Biotin-PEAC5-maleimide	Wako	Cat# 344-06391
N-Ethylmaleimide	Wako	Cat# 054-02063
TCEP Hydrochloride	Wako	Cat# 205-19863
(+/-)-Dithiothreitol	Wako	Cat# 048-29224
Urea	Wako	Cat# 213-00173
Catalase	Tokyo Chemical Industry	Cat# C0052
Trypsin (Sequencing grade)	Promega	Cat# V5113
25% trifluoroacetic acid solution	Wako	Cat# 203-12201
HRP-streptavidin	Sigma-Aldrich	Cat# RABHRP3
Acetonitrile	Wako	Cat# 014-00381
Formic acid	Wako	Cat# 063-05895

**Critical commercial assays**		

Pierce™ High pH Reversed-Phase Peptide Fractionation Kit	Thermo Scientific	Cat# 84868
Ultrafree Centrifugal filter	Millipore	Cat# UFC30SV00
Aurora Ultimate CSI 25 × 75 C18 UHPLC column	Ionopticks	Cat# AUR3-25075C18-CSI

**Deposited data**		

Proteomic Data Repository	jPOST	JPST003198

**Experimental models: Organisms/strains**		

C57BL6/J	Sankyo Lab Service	

**Software**		

Proteome Discoverer	Thermo Scientific	RRID:SCR_014477
Image Lab software	Bio-Rad Laboratories, Inc.	RRID:SCR_014210
GraphPad Prism	GraphPad	RRID:SCR_002798

**Other**		

Q Exactive Plus Orbitrap mass spectrometry	Thermo Scientific	

### Experimental model and study participant details

#### Animals

C57BL/6J wild-type male mice were obtained from Sankyo Lab Service (Tokyo, Japan). The mice ate a standard rodent food diet CRF-1 containing 0.45 mg/kg of selenium (Oriental Yeast, Tokyo, Japan). Mice were housed at 25°C on a 12-h light/12-h dark cycle with *ad libitum* access to water and food at the Animal Research Facility. To minimize the gender-related differences, only male mice aged 10 weeks, with a body weight of 20.7 ± 0.5 grams, were used in this study.

Three C57BL/6J male mice each were used for room temperature and cold exposure experiments, with no food but with water access during the experiments. Three single-caged mice kept in the standard mouse room were used for the room temperature group. Three single-caged mice were put into the cold chamber at 4°C for 3 hours for acute cold exposure. Immediately after the completion of the experiments, the mice were sacrificed to obtain the brown adipose tissue samples.

#### Ethical statement for animal experiments

All animal care and experiments were reviewed and approved by the ethical committee of Kanazawa University (Ethical Approval no. AP20-006).

### Method details

#### Tissue preparation

BAT samples were lysed using a chilled-lysis buffer containing catalase (1 mg), EDTA-free protease inhibitor (Roche), and 1× lysis buffer solution.^34^ Samples were homogenized using a pestle motor on ice until complete protein extraction was ensured. Samples were sonicated at 30 W and 20 kHz during 10 pulses on ice and then centrifuged at 15,000 g for 30 min at 4°C. Supernatants were collected, and the protein concentration was determined using a BCA assay.

#### Differential alkylation-labeling assay

Unless stated, all steps were performed in the dark. An extract (2 mg) of protein sample was treated with 24 mM N-ethylmaleimide (NEM; Fujifilm) in phosphate-buffered saline (pH= 7.3), with incubation at 37°C for 1 h, to label the free thiols. Protein was precipitated using methanol and chloroform^40^ and the resulting pellet containing the reversibly oxidized Cys-PTMs was resuspended in a 5mM tris(2-carboxyethyl) phosphine (TCEP; Fujifilm) buffer (pH= 7.8) to remove the reversibly oxidized Cys-PTMs for 30 min at 56°C, followed by 15 min at room temperature. Biotin-peac5-maleimide (BPM; Dojindo) (2.5 mM) was added to the previous buffer to label the reversibly oxidized Cys-PTMs for 1 h at 37°C. To ensure all cysteine residues were labeled, 5 mM TCEP buffer and 2.5 mM BPM were added to the previous solution and incubated for 1 h at 37°C. Labeled proteins were then precipitated with methanol and chloroform.

#### Trypsin digestion of labeled proteins

Digestion was performed as described previously.^11^ Briefly, trypsin digestion buffer containing 50 mM Tris-HCl (pH= 8.0), 4 mM 1,4 dithiothreitol (DTT, Fujifilm), and 8 M urea was added to the protein pellet. Labeled proteins were sonicated at 30 W and 20 kHz during 10 pulses on ice and then incubated at 60°C for 1 h. Next, samples were diluted with 50 mM Tris-HCl (pH= 8.0) to decrease the urea concentration to 1 M. The protein concentration was quantified using a BCA assay, and 300 μg of proteins were used together with sequencing-grade trypsin (Promega) in a protease: protein ratio of 1:60 (w/w) and incubated overnight at 37°C. To maximize the digestion, additional trypsin was added to a final protease: protein ratio of 1:60 (w/w) and incubated for 4 h at 37°C. Trypsin activity was stopped by adding 0.1% trifluoroacetic acid (TFA; Sigma-Aldrich) (pH= 1.0) to the digestion mix until the pH decreased to <4. Finally, labeled peptides were centrifuged at 20,000 g for 20 min at room temperature, and supernatants were collected in a new centrifuge and concentrated in a vacuum centrifuge for 18 h.

#### Liquid chromatography-mass spectrometry analysis

Dried labeled peptides, previously mentioned, were resuspended in 0.1% TFA in ultrapure water, and 100 μg of the sample was fractionated using a high pH reverse-phase peptide fractionation kit (Thermo Fisher Scientific). Briefly, the sample solution was added to a spin column and centrifuged, then the column was washed with ultrapure water, and the sample was eluted with 5 %, 10 %, 15 %, 20 %, and 50% acetonitrile (ACN; Wako Pure Chemical). Collected fractions were dried using a vacuum centrifuge and resuspended in 0.1% formic acid (FA; Wako Pure Chemical). Then, each fraction was column purified (UFC30SV00; Merk Millipore). Purified peptides were loaded and separated on the aurora column (25 cm × 75 μm, 1.7 μm C18; Ionopticks) with a linear ACN gradient (0%–40%) in 0.1% FA at a flow rate of 300 nL min-1. Peptide ions were detected using a Q Exactive Plus Orbitrap mass spectrometry (MS) (Thermo Fisher Scientific) in the data-dependent acquisition mode with the installed XcaliburTM software ver. 4.4 (Thermo Fisher Scientific). Full-scan mass spectra were acquired in the MS over 375-1,500 m/z with a resolution of 70,000. The MS/MS searches were conducted using SEQUEST HT search algorithms against the UniProt Mouse protein database using Proteome Discoverer (Version 3.0; Thermo Fisher Scientific). Five fractions (5%, 10%, 15%, 20%, and 50% ACN elutions) were analyzed as one sample. Protein identification was also performed with PD 3.0 using precursor ions quantifier nodes. The processing workflow included spectrum files RC, spectrum selector, SEQUEST HT search nodes, percolator, ptmRS, and minor feature-detector nodes. Methionine oxidation, Cysteine N-ethylmaleimide, and Cysteine Biotin-Peac5-maleimide were set as variable modifications, and mass tolerances in MS and MS/MS were set at 10 ppm and 0.6 Da, respectively. Trypsin was specified as the protease, and a maximum of two missed cleavages were allowed. Target-decoy database searches were used to calculate the false-discovery rate (FDR), and the peptide identification FDR was set at 1%.

#### Label-free quantification of proteins using mass spectrometry data

Label-free quantification was also performed with PD 3.0 using precursor ions quantifiler nodes. The consensus workflow included MSF files/Feature Mapper/precursor ion quantifier, and MSF Files/PSM groper/peptide validator, peptide and protein filter, protein scorer, protein marker, protein FDR validator, protein grouping, peptide in protein. Normalization of the abundances was performed using total peptide amount mode. The significant change of protein abundance was set by log2 fold change cut off 0 with *p* value < 0.05. The protein abundance data are plotted as volcano plot (Figure 1E) by using R version 4.4.2 with “ggpubr” version 0.6, “ggplot2” version 3.5.1, and “ggrepel” version 0.9.6 packages.

#### Cysteine proteome profile

The proteins considered for the cysteine identification analysis were only those with high and medium protein FDR confidence. Then, cysteine residues identified in each sample were classified according to their status: oxidized (BPM-tagged; +548.28), reduced (NEM-tagged; +125.05), and unlabeled, and the number of proteins was quantified per status. Next, all peptide sequences containing cysteine were given the same 15 amino acid length, aligning the cysteine in the center of the peptide. Also, the position of the cysteine residue was identified from their corresponding proteins. For calculating the % cysteine redox status in each group, the numbers of oxidized, reduced, and unlabeled cysteine residues were divided from the total number of cysteine identified. All labeled peptides were used to build a Venn diagram based on the cysteine redox status and condition.

#### Subcellular localization

All cysteine residue sites identified were matched with subcellular location information from the UniProKB database.^41^ All cysteine residue sites with multiple subcellular localization were considered for quantification. For calculating the % cysteine redox status per localization, the number of oxidized and reduced cysteine residues was divided into the total number of cysteine residues identified in the corresponding localization.

#### Reproducible cysteine residue sites identification

The total number of oxidized and reduced cysteine residues per group was evaluated according to their cysteine position. The cysteine positions detected in two of the three biological replicates under the same redox category were considered reproducible cysteine residue sites. If a protein has a reproducible cysteine residue site that appears multiple times in the exact category, this was counted only once to quantify the reproducible proteins.

#### Sequence analysis of the reactive cysteine residue sites

The amino acid sequences containing reactive cysteine residue sites of *M. musculus* were used as a query against the amino acid sequences of *H. sapiens* and *B. taurus* and performed multiple sequence alignment (MUSCLE) by using Clustal Omega.^42^ The alignment of each cysteine sought was highlighted manually, and the adjacent regions to the cysteine were manually cropped.

#### Enrichment pathway analyses

Gene Ontology (GO) and Kyoto Encyclopedia of Genes and Genomes (KEGG) enrichment pathway analyses were performed by subjecting the proteins belonging to cysteine residue sites independently to a GO category and KEGG pathway analysis using the ProteINSIDE.^43^

#### Motif analyses

Analysis of proximal amino acids was accomplished by extracting peptide sequences with a length of 15 amino acids centered around each labeled cysteine residue site. The pLogo algorithm^44^ was used to visualize the inputted aligned amino acid sequences. The foreground contains intended cysteine residue sites, and the background used was the protein mouse proteomic data set. Foreground peptide sequences were not subtracted from the background. The amino acid residues with a position weight matrix (PWM) score ≥ 3.75 were considered significantly enriched at the position localized relative to the reduced or oxidized cysteine residue.

The PWM values obtained from the pLogo algorithm were plotted in a heat map using R version 4.4.2 with “tidyverse” version 2.0.0 and “ggplot2” version 3.4.3 packages. The amino acid order used in the heat map was organized based on their properties.

#### Validation of differential alkylation method

The Cys-PTMs sites that were previously documented in the literature and were also identified in this study were validated by using the individual spectra of the detected peptides in Figure S2. The citations are as follows: Mic19,^45^ Slc25a20,^46^^,^^47^ Actin,^48^ DJ-1,^49^ Trx1.^50^

#### pKa analyses of reactive cysteine residues

The pKa value of reactive cysteine residues are calculated by using the python-based PROPKA3 program^51^ using the respective protein structure available in UniProKB database.

#### Disulfide bond dihedral angle energy of reactive cysteine residues

The disulfide bond dihedral angle energy values of reactive cysteine residues are calculated by using the web-based server (https://services.mbi.ucla.edu/disulfide/) using the respective protein structure available in UniProKB database.

#### Immunoprecipitation and western blot analysis

For MIC19 biotinylation status, the labeled proteins are immunoprecipitated by using Dynabeads™ Protein G immunoprecipitation kit (Invitrogen, 100007D) with MIC19 antibody (ThermoFisher, A305-497A). Then, we performed western blotting, as previously reported,^18^ and immunoblotted by HRP-streptavidin (Sigma-Aldrich, RABHRP3) in 1:5000 dilution.

### Quantification and statistical analysis

All data were analyzed using the GraphPad Prism 10 software. The bar graphs of Figures 1B–1D were shown with mean±SEM. Statistical methods were not used to determine the sample size. Statistical differences between the two groups were assessed using unpaired two-tailed student t-tests.

## Supplemental information

Document S1. Figures S1–S6

Table S1. Protein abundance in BAT under cold exposure compared to RT, related to Figure 1E

Table S2. Enriched KEGG pathways related to proteins of BAT increased under cold exposure based on Figure 1E, related to Figure 1F

Table S3. Enriched KEGG pathways related to proteins of BAT decreased under cold exposure based on Figure 1E, related to Figure 1F

Table S4. Biological processes enriched in GO analysis observed in reactive cysteine residues in BAT under RT and Cold groups, related to Figure 2D

Table S5. Molecular functions enriched in GO analysis observed in reactive cysteine residues in BAT under RT and Cold groups, related to Figure 2E

Table S6. Cellular compartments enriched in GO analysis observed in reactive cysteine residues in BAT under RT and Cold groups, related to Figure 2F

Table S7. Enriched KEGG pathways observed in reactive cysteine residues in BAT under RT and Cold groups, related to Figure 2G

Table S8. Structural and chemical properties of reactive cysteine residues, related to Figure S5

Table S9. Structural and chemical properties of non-reactive cysteine residues, related to Figure S5

Table S10. Biological processes enriched in GO analysis observed in exclusively found cysteine residues in BAT under RT, related to Figure S6A

Table S11. Molecular functions enriched in GO analysis observed in exclusively found cysteine residues in BAT under RT, related to Figure S6B

Table S12. Cellular compartments enriched in GO analysis observed in exclusively found cysteine residues in BAT under RT, related to Figure S6C

Table S13. Biological processes enriched in GO analysis observed in exclusively found cysteine residues in BAT under cold exposure, related to Figure S6D

Table S14. Molecular functions enriched in GO analysis observed in exclusively found cysteine residues in BAT under cold exposure, related to Figure S6E

Table S15. Cellular compartments enriched in GO analysis observed in exclusively found cysteine residues in BAT under cold exposure, related to Figure S6F

Table S16. Enriched KEGG pathways observed in exclusively found cysteine residues in BAT under RT, related to Figure 3A

Table S17. Enriched KEGG pathways observed in exclusively found cysteine residues in BAT under cold exposure, related to Figure 3B
