# Supplementary material for: Cysteine redoxome landscape in mouse brown adipose tissue under acute cold exposure
Source: iScience. 2025 Feb 17;28(3):112051. doi: 10.1016/j.isci.2025.112051 (PMC11915156; doi:10.1016/j.isci.2025.112051)
Supplement: Document S1. Figures S1–S6 [file mmc1.pdf]

iScience, Volume *28*

Supplemental information

Cysteine redoxome landscape in mouse brown adipose tissue under acute cold exposure

Hein Ko Oo, Cynthia M. Galicia-Medina, Takumi Nishiuchi, Ryota Tanida, Hisanori Goto, Yujiro Nakano, Yumie Takeshita, Yoshiro Saito, Hiroaki Takayama, and Toshinari Takamura

Room Temperature
(3 samples)

Redox status	Quantified cysteines
Oxidized-Cys	462
Reduced-Cys	3900

Cold exposure
(3 samples)

Redox status	Quantified cysteines
Oxidized-Cys	464
Reduced-Cys	1862

Cysteine residue sites comfirmed
at least in 2 samples

Redox status	Quantified cysteines
Oxidized-Cys	118
Reduced-Cys	1097

Redox status	Quantified cysteines
Oxidized-Cys	103
Reduced-Cys	569

Supplementary Figure 1. Criteria for classifying reproducible cysteine residues.
A schematic representation illustrates the selection process of reproducible cysteine residues, validated across triplicate samples within each experimental condition. From all labeled cysteine residues, those identified consistently in at least two samples were designated as reproducible cysteine sites for future comparison between experimental conditions.

A

Accession ID	Protein Name	Peptide Sequence	Cys position	Reference
Q9CRB9	MICOS complex subunit Mic19	YEYHPV[C]ADLQTK	183	45
Q7TPW6	Mitochondrial carnitine/acylcarnitine translocase (Slc25a20)	YSGTLD[C]AK	155	46, 47
P63260	Actin, cytoplasmic	CPEALFQPSFLGMES[C]GIHETTFNSIMK	272	48
Q99LX0	Protein DJ-1	DVMI[C]PDTSLEDAK	53	49
P10639	Thioredoxin 1 (Trx1)	[C]MPTFQFYK	73	50

B

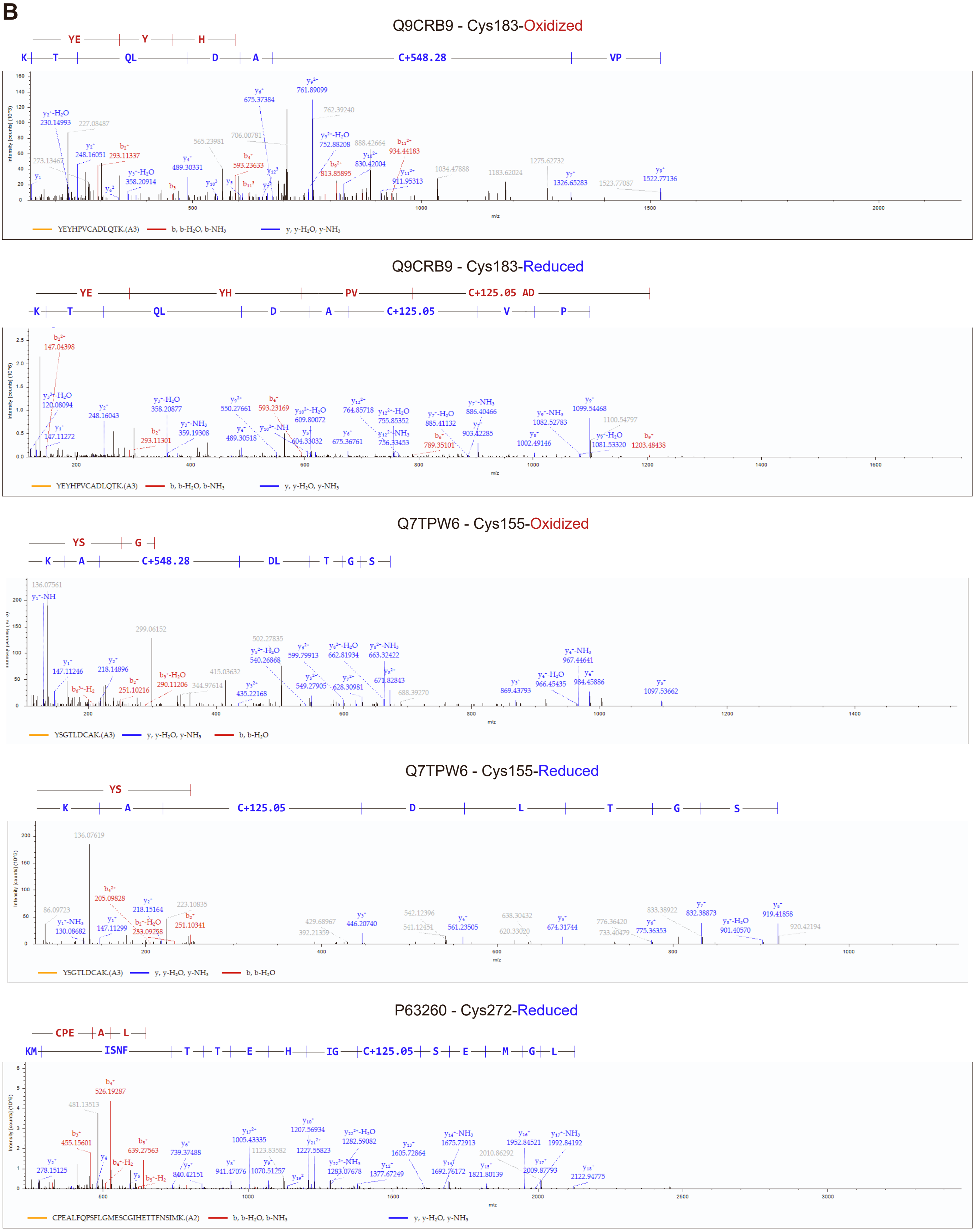

P10639 - Cys73-Reduced

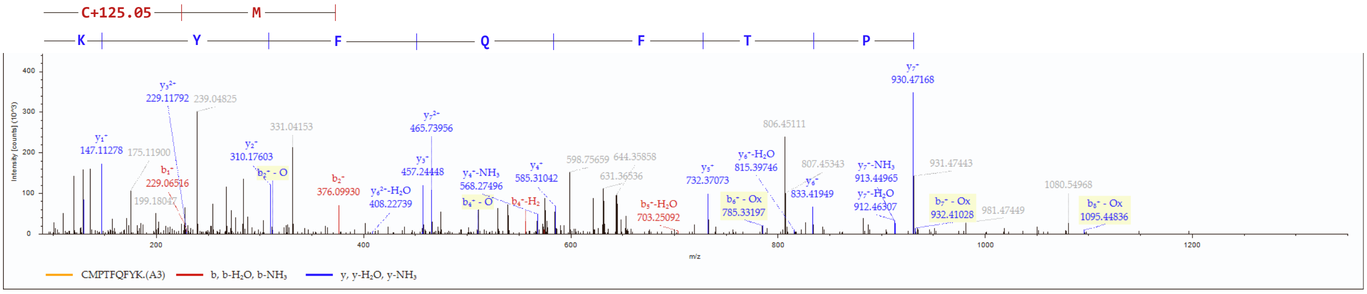

P10639 - Cys73-Oxidized

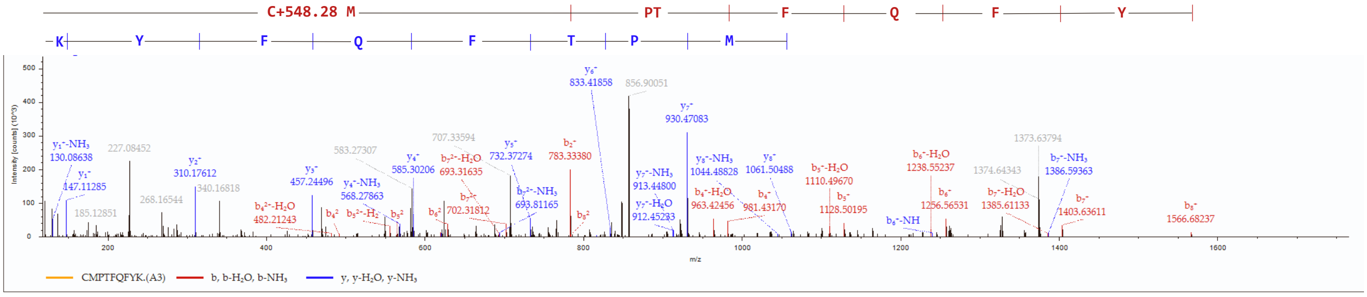

Q99LX0 - Cys53-Reduced

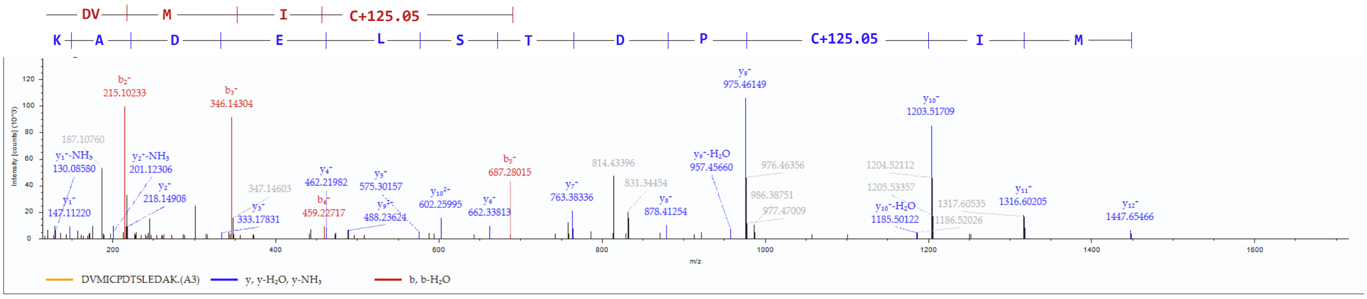

Q99LX0 - Cys53-Oxidized

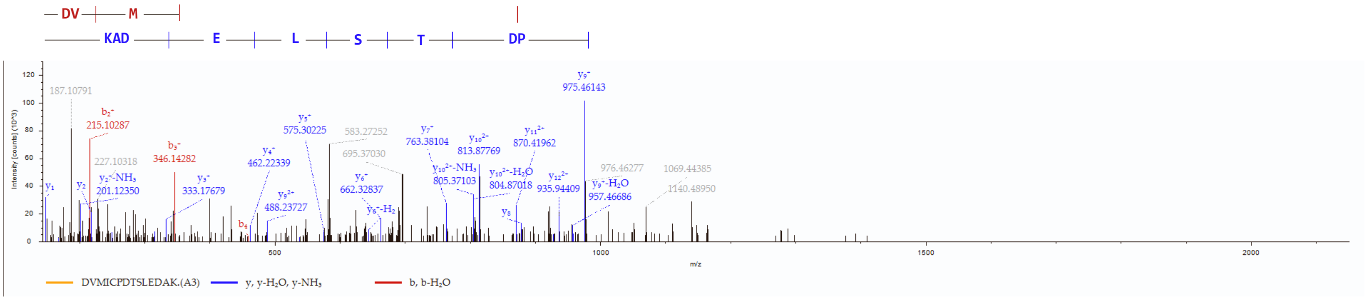

Supplementary Figure 2. Validation of the differential alkylation method.
(A) Table showing Cys-PTMs sites that were previously documented in the literature and were also identified in this study. (B) Individual spectra of the peptides described in (A). C+125 corresponds to a Cys residue alkylated with N-ethylmaleimide (NEM), thus, a Cys that was reduced in cells. C+548.28 corresponds to a Cys residue alkylated with biotin-PECA5-maleimide (BPM), thus, a Cys that was oxidized in cells. The fragment picks of both the b and y ions are shown in the spectra in red and blue respectively. All MS/MS spectra were visualized with Protein Discoverer version 3.0.

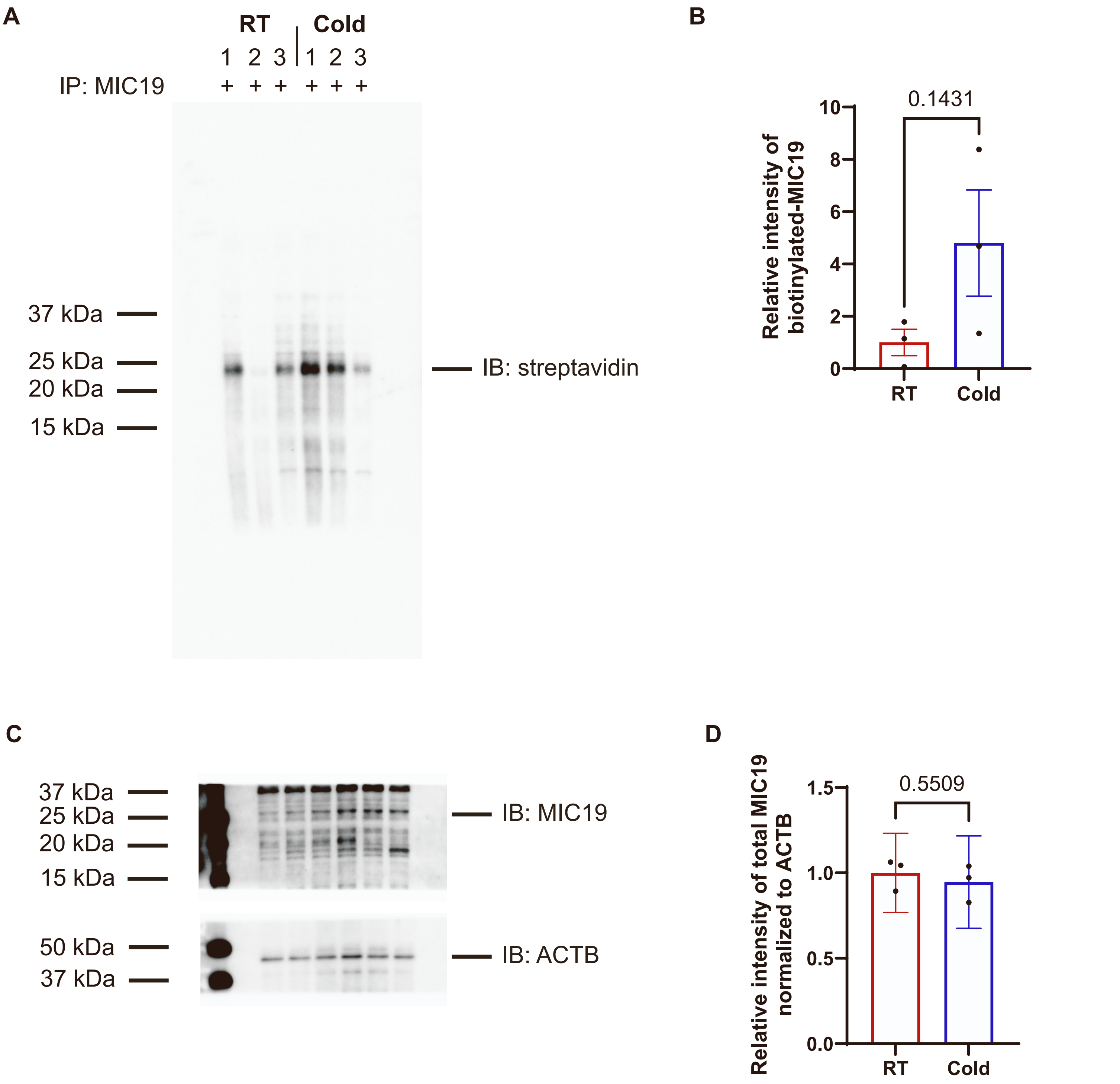

Supplementary Figure 3. Western blot image of MIC19 biotinylation status.
(A and B) Full western blot image (A) and densitometric analysis (B) of MIC19 biotinylation status. The BAT tissue lysates were labeled with NEM and BPM as described in Fig. 1 step 1 to 4. 5 ug of labeled proteins were used for immuprecipitation by anti-mouse MIC19 antibody, then loaded to SDS-PAGE and immunoblotted by streptavidin.
(C and D) Full western blot images (C) and densitometric analysis (D) of MIC19 protein level in each sample of (A) without immunoprecipitation. The data in (B) and (D) are shown as mean ± SEM. Statistical significance is calculated by two-tailed unpaired student’s t-test.

NADH dehydrogenase 1 beta subcomplex subunit 10 (Ndufb10) – C77

```
sp|O96000|NDUBA_HUMAN      YYYYHRQYRRVPDITECKEEDIMCMYEAEMQWKRDYKVDQEIINIMQDRLKACQQREGQN   114
sp|Q9DCS9|NDUBA_MOUSE      TYYYHRQYRRVPDITECKEGDVLCIYEAEMQWRRDFKVDQEIMNIIQERLKACQQREGEN   120
sp|Q02373|NDUBA_BOVIN      YYYYHREFRRVPDITECQEKDVLCMFEAEMQWRRDYKVDQEIVNIIQERLKACQQREGES   120
                            *****::*********:* *::*::******:**:******:**:*:**********:.
```

MICOS complex subunit Mic19 (Chchd3) – C183

```
sp|Q5E9D3|MIC19_BOVIN      PVCADLQAQILQCYRQNTQQTLSCSALASQYMRCVNQAKQSTLEKGG 227
sp|Q9CRB9|MIC19_MOUSE      PVCADLQTKILQCYRQNTQQTLSCSALASQYMHCVNHAKQSMLEKGG 227
sp|Q9NX63|MIC19_HUMAN      PVCADLQAKILQCYRENTHQTLKCSALATQYMHCVNHAKQSMLEKGG 227
                           *******::******:**:***.*****:***:***:**** *****
```

Elongation factor 1-alpha 1 (Eef1a1) – C411

```
sp|P10126|EF1A1_MOUSE      LDCHTAHIACKFAELKEKIDRRSGKKLEDGPKFLKSGDAAIVDMVPGKPMCVESFSDYPP   420
sp|P68104|EF1A1_HUMAN      LDCHTAHIACKFAELKEKIDRRSGKKLEDGPKFLKSGDAAIVDMVPGKPMCVESFSDYPP   420
sp|P68103|EF1A1_BOVIN      LDCHTAHIACKFAELKEKIDRRSGKKLEDGPKFLKSGDAAIVDMVPGKPMCVESFSDYPP   420
                           ************************************************************
```

NADH dehydrogenase 1 alpha subcomplex subunit 8 (Ndufa8_ - C70

```
sp|Q9DCJ5|NDUA8_MOUSE      KLVNGCALNFFRQIKSHCAEPFTEYWTCLDYSNMQLFRHCRQQQAKFDQCVLDKLGWVRP   120
sp|P51970|NDUA8_HUMAN      KLVNKCALDFFRQIKRHCAEPFTEYWTCIDYTGQQLFRHCRKQQAKFDECVLDKLGWVRP   120
sp|P42029|NDUA8_BOVIN      KLVNQCALEFFRQIKRHCAEPFTEYWTCIDYSGLQLFRRCRKQQAQFDECVLDKLGWVRP   120
                           **** ***:****** ************:**:. ****:**:***:**:***********
```

Selenium-binding protein 1 (Selenbp1) – C8

```
sp|P17563|SBP1_MOUSE      MATKCTKCGPGYSTPLEAMKGPREEIVYLPCIYRNTGTEAPDYLATVDVDPKSPQYSQVI    60
sp|Q13228|SBP1_HUMAN      MATKCGNCGPGYSTPLEAMKGPREEIVYLPCIYRNTGTEAPDYLATVDVDPKSPQYCQVI    60
sp|Q2KJ32|SBP1_BOVIN      MATKCGKCGPGYPSPLEAMKGPREELVYLPCIYRNTGTEAPDYLATVDVNPKSPQYSQVI    60
                          ***** :***** :***********:***********************:******.***
```

Hemopexin (Hpx) – C230

```
sp|Q3SZV7|HEMO_BOVIN      KRSWPAVGNCSSAIRWLNRYYCFRGNKFLRFDPVTGEVNSTYPRDVRDYFMSCPNRGHAH   239
sp|Q91X72|HEMO_MOUSE      ERSWSTVGNCTAALRWLERYYCFQGNKFLRFNPVTGEVPPRYPLDARDYFVSCPGRGHGR   237
sp|P02790|HEMO_HUMAN      ERSWPAVGNCSSALRWLGRYYCFQGNQFLRFDPVRGEVPPRYPRDVRDYFMPCPGRGHGH   238
                          :*** :****::*:*** *****:**:****:** ***   ** *.****: **.***.:
```

Basigin (Bsg) – C203

```
sp|P18572|BASI_MOUSE      DTLPDLHTKYIVDADDRSGEYSCIFLPEPVGRSEINVEGPPRIKVGKKSEHSSEGELAKL   240
sp|P35613|BASI_HUMAN      DALPGQKTEFKVDSDDQWGEYSCVFLPEPMGTANIQLHGPPRVKAVKSSEHINEGETAML   240
sp|Q865R3|BASI_BOVIN      DALPGQKTEFKVDSDDQWGEYSCVFLPEPMGTANIQLHGPPRVKAVKSSEHINEGETAML   124
                          *:**. :*:: **:**: *****:*****:* ::*::.****:*. *.*** .*** * *
```

Alpha-2-HS-glycoprotein (Fetuin-A) (Ahsg) – C219

```
sp|P02765|FETUA_HUMAN      QLEEISRAQLVPLPPSTYVEFTVSGTDCVAKEATEAAKCNLLAEKQYGFCKATLS-EKLG   239
sp|P29699|FETUA_MOUSE      KLVEISRAQNVPLPVSTLVEFVIAATDCTAKEVTDPAKCNLLAEKQHGFCKANLM-HNLG   239
sp|P12763|FETUA_BOVIN      QLVEISRAQFVPLPVSVSVEFAVAATDCIAKEVVDPTKCNLLAEKQYGFCKGSVIQKALG   240
                           :* ****** **** *. ***.::.*** ***..: :*********:****..:  . **
```

COX assembly mitochondrial protein (Cmc1) – C57

```
tr|Q9CVE7|Q9CVE7_MOUSE      EVALRLSRPRLRASRRLRAQPSAGSSPPGPLAQQHLRHVEKDVLIPKIIREKARERCSEQ  60
sp|Q7Z7K0|COXM1_HUMAN       --------------------------MALDPADQHLRHVEKDVLIPKIMREKAKERCSEQ  34
sp|Q3SZM6|COXM1_BOVIN       --------------------------MALDPSEQHLRHVEKDVLIPKIMREKARERCSEQ  34
                                                        ::***************:****:******
```

Membrane primary amine oxidase (Aoc3) – C430

```
sp|O70423|AOC3_MOUSE      APKTLRDAFCVFEQNQGLPLRRHHSDFYSHYFGGVVGTVLVVRSVSTLLNYDYIWDMVFH   480
sp|Q16853|AOC3_HUMAN      APKTIRDAFCVFEQNQGLPLRRHHSDLYSHYFGGLAETVLVVRSMSTLLNYDYVWDTVFH   480
sp|Q9TTK6|AOC3_BOVIN      APRTLHDAFCVFEQNKGLPLRRHHSDFISQYFGGVVETVLVFRSVSTLLNYDYVWDMVFH   480
                          **:*::*********:**********: *:****:. ****.**:********:** ***
```

Perilipin-1 (Plin1) – C55

```
sp|Q8CGN5|PLIN1_MOUSE       MSMNKGPTLLDGDLPEQENVLQRVLQLPVVSGTCECFQKTYNSTKEAHPLVASVCNAYEK  60
sp|O60240|PLIN1_HUMAN       MAVNKGLTLLDGDLPEQENVLQRVLQLPVVSGTCECFQKTYTSTKEAHPLVASVCNAYEK  60
tr|A4IFB3|A4IFB3_BOVIN      MAVNKGPTLLDGDLPEQENVLQRVLQLPVVSGTCECFQKTYASTKEAHPLVASVCNAYEK  60
                            *::*** ********************************** *****************
```

Serotransferrin (Tf) – C28, 67, 260, 363, 386, 395

```
sp|Q29443|TRFE_BOVIN      MRPAVRALLACAVLGLCLADPERTVRWCTISTHEANKCASFRENVLRIL-ESGPFVSCVK   59
sp|Q921I1|TRFE_MOUSE      MRLTVGALLACAALGLCLAVPDKTVKWCAVSEHENTKCISFRDHMKTVLPPDGPRLACVK   60
sp|P02787|TRFE_HUMAN      MRLAVGALLVCAVLGLCLAVPDKTVRWCAVSEHEATKCQSFRDHMKSVIPSDGPSVACVK   60
                          ** :* ***.**.****** *::**:**::* ** .** ***:::  ::  .** ::***

sp|Q29443|TRFE_BOVIN      KTSHMDCIKAISNNEADAVTLDGGLVYEAGLKPNNLKPVVAEFHGTKDNPQTHYYAVAVV   119
sp|Q921I1|TRFE_MOUSE      KTSYPDCIKAISASEADAMTLDGGWVYDAGLTPNNLKPVAAEFYGSVEHPQTYYYAVAVV   120
sp|P02787|TRFE_HUMAN      KASYLDCIRAIAANEADAVTLDAGLVYDAYLAPNNLKPVVAEFYGSKEDPQTFYYAVAVV   120
                          *:*: ***:**: .****:***.* **:* * *******.***:*: :.***.*******

sp|Q29443|TRFE_BOVIN      PEDRKNYELLCGDNTRKSVDDYQECYLAMVPSHAVVARTVGGKEDVIWELLNHAQEHFGK   299
sp|Q921I1|TRFE_MOUSE      KADRDQYELLCLDNTRKPVDQYEDCYLARIPSHAVVARKNNGKEDLIWEILKVAQEHFGK   295
sp|P02787|TRFE_HUMAN      KADRDQYELLCLDNTRKPVDEYKDCHLAQVPSHTVVARSMGGKEDLIWELLNQAQEHFGK   295
                            **.:***** ***** **:*::*:** :***:****. .****:***:*: *******

sp|Q29443|TRFE_BOVIN      DEC-MVKWCAIGHQERTKCDRWSGFSGGAIECETAENTEECIAKIMKGEADAMSLDGGYL   418
sp|Q921I1|TRFE_MOUSE      D-NSPVKWCALSHLERTKCDEWSIISEGKIECESAETTEDCIEKIVNGEADAMTLDGGHA   414
sp|P02787|TRFE_HUMAN      DECKPVKWCALSHHERLKCDEWSVNSVGKIECVSAETTEDCIAKIMNGEADAMSLDGGFV   415
                          *    *****:.* ** ***.**  * * *** :**.**:** **::******:****.
```

NADH dehydrogenase iron-sulfur protein 5 (Ndufs5) – C108

```
sp|Q02379|NDUS5_BOVIN             -----MPFFDVQKRLGVDLDRWMTIQSAEQPHKIPSRCHAFEKEWIECAHGIGSIRAEKE   55
tr|A0A1S6GWI4|A0A1S6GWI4_MOUSE    CSFTAMPFLDIQKKLGISLDRHFMFLSAEQPYKNAARCHAFEKEWIECAHGIGGTRAKKE   120
sp|O43920|NDUS5_HUMAN             -----MPFLDIQKRFGLNIDRWLTIQSGEQPYKMAGRCHAFEKEWIECAHGIGYTRAEKE   55
                                       ***:*:**::*:.:** : : *.***:*  .****************  **:**
```

Albumin (Alb) – C77, 86, 461, 462, 500, 501, 511

```
tr|Q546G4|Q546G4_MOUSE      DEHAKLVQEVTDFAKTCVADESAANCDKSLHTLFGDKLCAIPNLRENYGELADCCTKQEP   120
sp|P02768|ALBU_HUMAN        EDHVKLVNEVTEFAKTCVADESAENCDKSLHTLFGDKLCTVATLRETYGEMADCCAKQEP   120
sp|P02769|ALBU_BOVIN        DEHVKLVNELTEFAKTCVADESHAGCEKSLHTLFGDELCKVASLRETYGDMADCCEKQEP   120
                            ::*.***:*:*:**********  .*:*********:** : .***.**::**** ****

tr|Q546G4|Q546G4_MOUSE      KLGEYGFQNAILVRYTQKAPQVSTPTLVEAARNLGRVGTKCCTLPEDQRLPCVEDYLSAI   480
sp|P02768|ALBU_HUMAN        QLGEYKFQNALLVRYTKKVPQVSTPTLVEVSRNLGKVGSKCCKHPEAKRMPCAEDYLSVV   480
sp|P02769|ALBU_BOVIN        KLGEYGFQNALIVRYTRKVPQVSTPTLVEVSRSLGKVGTRCCTKPESERMPCTEDYLSLI   479
                            :**** ****::****:*.**********.:*.**:**::**. ** :*:**.***** :

tr|Q546G4|Q546G4_MOUSE      LNRVCLLHEKTPVSEHVTKCCSGSLVERRPCFSALTVDETYVPKEFKAETFTFHSDICTL   540
sp|P02768|ALBU_HUMAN        LNQLCVLHEKTPVSDRVTKCCTESLVNRRPCFSALEVDETYVPKEFNAETFTFHADICTL   540
sp|P02769|ALBU_BOVIN        LNRLCVLHEKTPVSEKVTKCCTESLVNRRPCFSALTPDETYVPKAFDEKLFTFHADICTL   539
                            **::*:*********::*****: ***:********  ******* *. : ****:*****
```

Cathepsin B (Ctsb) – C93

```
sp|P10605|CATB_MOUSE      TVLGGPKLPGRVAFGEDIDLPETFDAREQWSNCPTIGQIRDQGSCGSCWAFGAVEAISDR   120
sp|P07858|CATB_HUMAN      TFLGGPKPPQRVMFTEDLKLPASFDAREQWPQCPTIKEIRDQGSCGSCWAFGAVEAISDR   120
sp|P07688|CATB_BOVIN      AILGGPKLPQRDAFAADVVLPESFDAREQWPNCPTIKEIRDQGSCGSCWAFGAVEAISDR   120
                          :.***** * *  *  *: ** :******* :**** :**********************
```

Cytochrome c oxidase subunit 6B1 (Cox6b1) – C54

```
sp|P56391|CX6B1_MOUSE      MAEDIKTKIKNYKTAPFDSRFPNQNQTKNCWQNYLDFHRCEKAMTAKGGDVSVCEWYRRV   60
sp|P00429|CX6B1_BOVIN      MAEDIQAKIKNYQTAPFDSRFPNQNQTRNCWQNYLDFHRCEKAMTAKGGDVSVCEWYRRV   60
sp|P14854|CX6B1_HUMAN      MAEDMETKIKNYKTAPFDSRFPNQNQTRNCWQNYLDFHRCQKAMTAKGGDISVCEWYQRV   60
                           ****:::*****:**************:************:*********:******:**
```

Trifunctional enzyme subunit alpha, mitochondrial (Hadha) - 470

```
sp|O46629|ECHB_BOVIN      ------------------------------------------------------------   325
sp|Q8BMS1|ECHA_MOUSE      LTSFERDSIFSNLIGQLDYKGFEKADMVIEAVFEDLGVKHKVLKEVESVTPEHCIFASNT   476
sp|P40939|ECHA_HUMAN      LTSFERDSIFSNLTGQLDYQGFEKADMVIEAVFEDLSLKHRVLKEVEAVIPDHCIFASNT   476
```

Solute carrier family 25 (Mitochondrial carnitine/acylcarnitine translocase) (Slc25a20) – C155

```
tr|Q7TPW6|Q7TPW6_MOUSE      GVFTTGIMTPGERIKCLLQIQASSGENKYSGTLDCAKKLYQEFGIRGFYKGTVLTLMRDV   180
sp|O43772|MCAT_HUMAN        GVFTTGIMTPGERIKCLLQIQASSGESKYTGTLDCAKKLYQEFGIRGIYKGTVLTLMRDV   180
tr|Q3SZA4|Q3SZA4_BOVIN      GVFTTGIMTPGERIKCLLQIQASSGETKYTGPLDCAKKLYKEAGVRGIYKGTVLTLMRDV   180
                            *************************.**:* ********:* *:**:************
```

Aconitate hydratase, mitochondrial (Aco2) – C126

```
sp|Q99798|ACON_HUMAN      PSTIHCDHLIEAQVGGEKDLRRAKDINQEVYNFLATAGAKYGVGFWKPGSGIIHQIILEN    180
sp|Q99KI0|ACON_MOUSE      PSTIHCDHLIEAQVGGEKDLRRAKDINQEVYNFLATAGAKYGVGFWRPGSGIIHQIILEN    180
sp|P20004|ACON_BOVIN      PSTIHCDHLIEAQLGGEKDLRRAKDINQEVYNFLATAGAKYGVGFWRPGSGIIHQIILEN    180
                          *************:********************************:*************
```

Calpastatin (Calpain inhibitor) (Cast) – C408

```
sp|P51125|ICAL_MOUSE      KCGEDEDTVPAEYRLKPAKDKDGKPLLPEPEETSKSLSESELIGELSADFDRSTYQDKPS    466
sp|P20810|ICAL_HUMAN      KCGEDDETIPSEYRLKPATDKDGKPLLPEPEEKPKPRSESELIDELSEDFDRSECKEKPS    386
sp|P20811|ICAL_BOVIN      KCGEDEETVPSEYRLKPATDKDGKPLLPEAEEKPKPLSESELIDELSEDFDQSKPTEKQS    374
                          *****::*:*:*******.********** **. *  ******.*** ***:*   :* *
```

Elongation factor 1-gamma (Eef1g) – C266

```
sp|Q3SZV3|EF1G_BOVIN      KLKPQAERKEGKEEKKAAAPAPEEELDECEQALAAEPKAKDPFAHLPKSTFVLDEFKRKY    300
sp|Q9D8N0|EF1G_MOUSE      KQKPQAERK---EEKKAAAPAPEEEMDECEQALAAEPKAKDPFAHLPKSTFVLDEFKRKY    297
sp|P26641|EF1G_HUMAN      KQKPQAERK---EEKKAAAPAPEEEMDECEQALAAEPKAKDPFAHLPKSTFVLDEFKRKY    297
                          * *******   *************:**********************************
```

Enoyl-CoA hydratase, mitochondrial (Echs1) – C111

```
sp|Q58DM8|ECHM_BOVIN      LCNGLIVELNQALQAFEEDPAVGAIVLTGGEKVFAAGADIKEMQSLTFQNCYSGGFLSHW    120
sp|Q8BH95|ECHM_MOUSE      LCNGLIEELNQALETFEQDPAVGAIVLTGGDKAFAAGADIKEMQNRTFQDCYSSKFLSHW    120
sp|P30084|ECHM_HUMAN      LCDGLIDELNQALKTFEEDPAVGAIVLTGGDKAFAAGADIKEMQNLSFQDCYSSKFLKHW    120
                          **:*** ******::**:************:*.***********. :**:***. **.**
```

Supplementary Figure 4. Alignment of reactive cysteine residues across mouse, human, and bovine. Amino acid sequences were aligned using the multiple sequence alignment (MUSCLE) analysis, comparing *M. musculus* with *H. sapiens* and *B. taurus*.

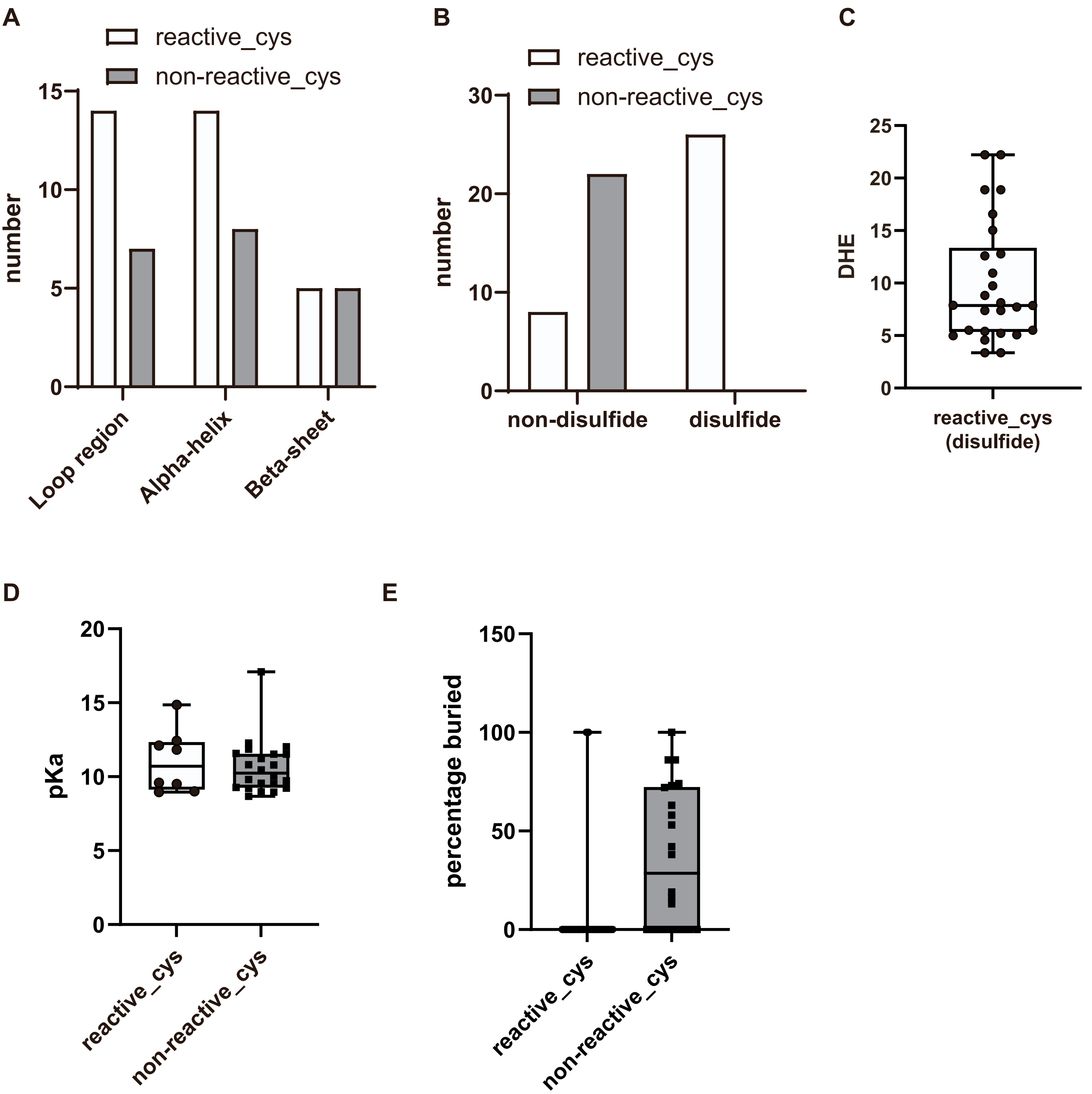

Supplementary Figure 5. Structural and chemical properties of reactive cysteine residues.
(A) Structural location of reactive cysteine residues in their respective protein structure compared to the non-reactive cysteine residues with nearby lysine residues in Region I of Fig. 2A. (B) The nature of reactive and non-reactive cysteine residues from (A); disulfide, cysteine residue involved in intramolecular disulfide bond formation; non-disulfide, cysteine residue not involved in intramolecular disulfide bond formation. (C) Dihedral energy of disulfide bonds in which reactive cysteine residues are involved. (D and E) The pKa (D) and buried percentage (E) of reactive and non-reactive cysteine residues according to PROPKA 3 analysis.

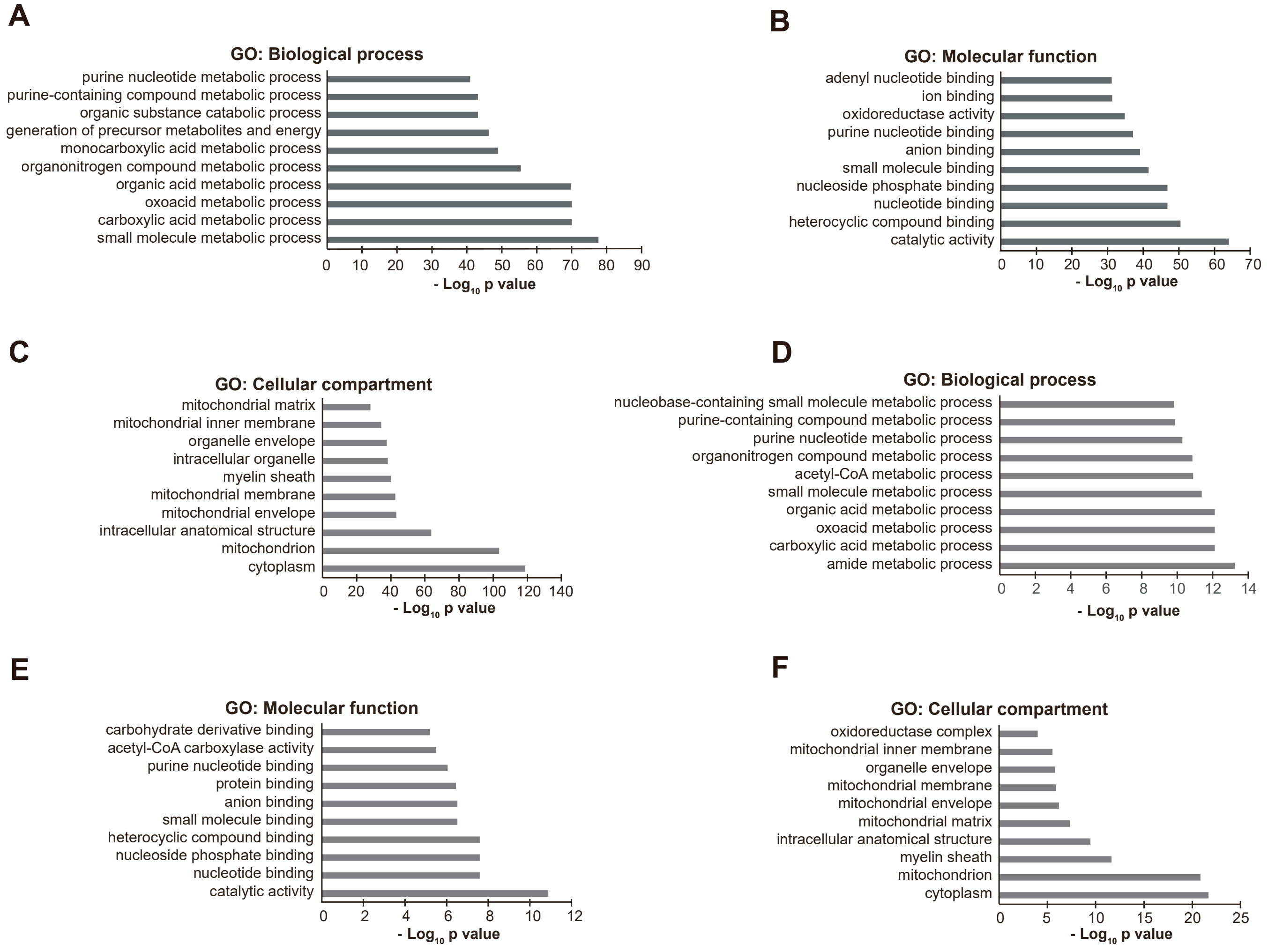

Supplementary Figure 6. Identifying Gene ontology (GO) terms associated with exclusive cysteine residues in RT and cold conditions.
A-C: GO enrichment analyses based on biological processes (A), molecular functions (B), and cellular compartments (C) exclusively found in the cysteine residues under RT. D-F: GO enrichment analyses based on biological processes (A), molecular functions (B), and cellular compartments (C) exclusively identified in cysteine residues under cold exposure. Selected terms relevant to cellular homeostasis are presented. Complete lists of enriched terms can be found in Supplementary Tables 10-15.
